# Structural basis of a distinct α-synuclein strain that promotes tau inclusion in neurons

**DOI:** 10.1016/j.jbc.2025.108351

**Published:** 2025-02-25

**Authors:** Chuanqi Sun, Kang Zhou, Peter DePaola, Cally Li, Virginia M.Y. Lee, Z. Hong Zhou, Chao Peng, Lin Jiang

**Affiliations:** 1 Department of Neurology, David Geffen School of Medicine, University of California, Los Angeles (UCLA), Los Angeles, California, USA; 2 Departments of Biological Chemistry and Chemistry and Biochemistry, UCLA-DOE Institute, UCLA, Los Angeles, California, USA; 3 California NanoSystems Institute, UCLA, Los Angeles, California, USA; 4 Alsion Montessori High School, Fremont, California, USA; 5 Department of Pathology and Laboratory Medicine, Institute on Aging and Center for Neurodegenerative Disease Research, Philadelphia, Pennsylvania, USA; 6 Department of Microbiology, Immunology and Molecular Genetics, UCLA, Los Angeles, California, USA

**Keywords:** α-synuclein, tau, co-aggregation, Parkinson's disease, Alzheimer's disease

## Abstract

Amyloidoses are predominantly associated with the accumulation of persistent aggregates of a particular protein. For example, the protein α-synuclein characteristically aggregates in Parkinson's disease (PD), while amyloid beta and tau deposits are associated with Alzheimer's disease (AD). However, α-synuclein-positive inclusions have been reportedly found in some tauopathies, and *vice versa*; tau-positive inclusions can be found in synucleinopathies. This suggests that there may be coexistence or crosstalk between these proteinopathies. This coexistence suggests that the simultaneous presence of these misfolded proteins may amplify pathogenic mechanisms. However, the crosstalk between these two types of proteopathies remains poorly understood. We now determine the structure of α-synuclein fibrils that directly promote tau aggregation by cryogenic electron microscopy. Helical reconstruction at 2.6 Å resolution reveals a new α-synuclein fibril polymorph we term “strain B”; its core is unique, incorporating both the N- and C-termini of α-synuclein. The design of peptides meant to inhibit the formation of this structure demonstrates that the C-terminal domain fragment (D105-E115) of α-synuclein is critical for the formation of “strain B” fibrils and may play a key role in its interaction with tau. We hypothesize that the unique structure of pathological α-synuclein significantly contributes to tau co-aggregation and plays a role in the intricate interactions among Alzheimer's, Parkinson's, and other neurodegenerative diseases. These findings open new avenues for drug targeting, discovery, and improve our understanding of neurodegenerative pathology.

Neurodegenerative disorders broadly refer to a range of pathologies characterized by a progressive decline in neuronal structure and/or function, leading to deficits in cognitive function, and are often—but not exclusively—associated with movement problems (1, 2). The majority of neurological diseases are characterized by abnormal protein accumulation, and this trend is most clearly represented in Parkinson's and Alzheimer's, where α-syn and tau respectively form insoluble fibrils within neurons or glial cells, accumulating to form aggregations that appear as Lewy bodies (LBs) and neurofibrillary tangles (2, 3, 4).

α-syn is a 140-amino-acid protein mainly expressed in the presynaptic termini of neurons and plays a significant role in the trafficking of synaptic vesicles and the secretion of neurotransmitters (5, 6). Under physiological conditions, α-syn exists as a natively unfolded monomer or an α-helical tetramer (7). With abnormal conformational changes, α-syn can further self-assemble into β-sheet-rich oligomeric and fibrillar interspaced structures (8). A wide range of triggers, including oxidative stress, post-translational modifications, genetic mutations, and interactions with other proteins or lipids, have been identified as modulators of α-syn oligomerization and fibrillization (9). The transformation and aggregation of α-synuclein are characteristic causes underlying the pathogenesis of Parkinson's disease and other neurodegenerative disorders (10, 11).

Tau is a microtubule-associated protein primarily expressed in neurons (1, 12), whose primary function is the stabilization of microtubules and control of axonal transport. Six isoforms of tau exist, where each differs in the number of isoform N-terminal inserts itself, and microtubule-binding proceeds and repeats (8). It is noted that tau undergoes conformational changes from its native functional state, forming aggregates and eventually paired helical filaments and straight filaments, similar to α-syn (13). These conformational changes are similarly instigated by factors such as phosphorylation, truncation, glycation, and mutations within the protein (14, 15, 16).

Although α-synuclein has been the classic hallmark pathology in diseases like Parkinson's, there is increasing evidence that points towards cross-talk and co-aggregating conformational changes of α-syn and tau within mixed neurodegenerative diseases such as Dementia with Lewy Bodies (DLB), Alzheimer's disease with Lewy bodies, and Parkinson's disease with Dementia (9, 10, 11, 17, 18, 19, 20). A further proposition suggests that the co-aggregation of α-synuclein and tau results in a potentiation of neurotoxicity and disease progression by influencing each other's aggregation-clearance dynamics and physiological functions (10, 17). This co-accumulation leads to the propagation of pathology, where these pathogenic proteins spread akin to prions and move from one cell type to another within the brain or from one brain region to another (21, 22). Consequently, this propagation and translocation process escalates the burden and lethality of the pathology.

Here, we find that a recently established α-syn fibril strain, “B”, markedly prompts the co-aggregation of tau protein in both neuronal cells and *in vitro* settings (23). We use cryogenic electron microscopy (cryoEM) to determine the atomic structure of the strain, which led us to hypothesize that it presents a new interface for direct interaction with tau. We design inhibitors that target this region and mitigate the co-aggregation effect of α-syn on tau protein. Collectively, these findings suggest that a distinct region at the C-terminus of α-syn may play a pivotal role in tau protein aggregation. This provides a potential therapeutic direction and theoretical foundation for mitigating the progression of such diseases.

## Results

### Production of **α**-syn strain B significantly enhances tau inclusion in neurons and *in vitro* studies

**Figure 1:**
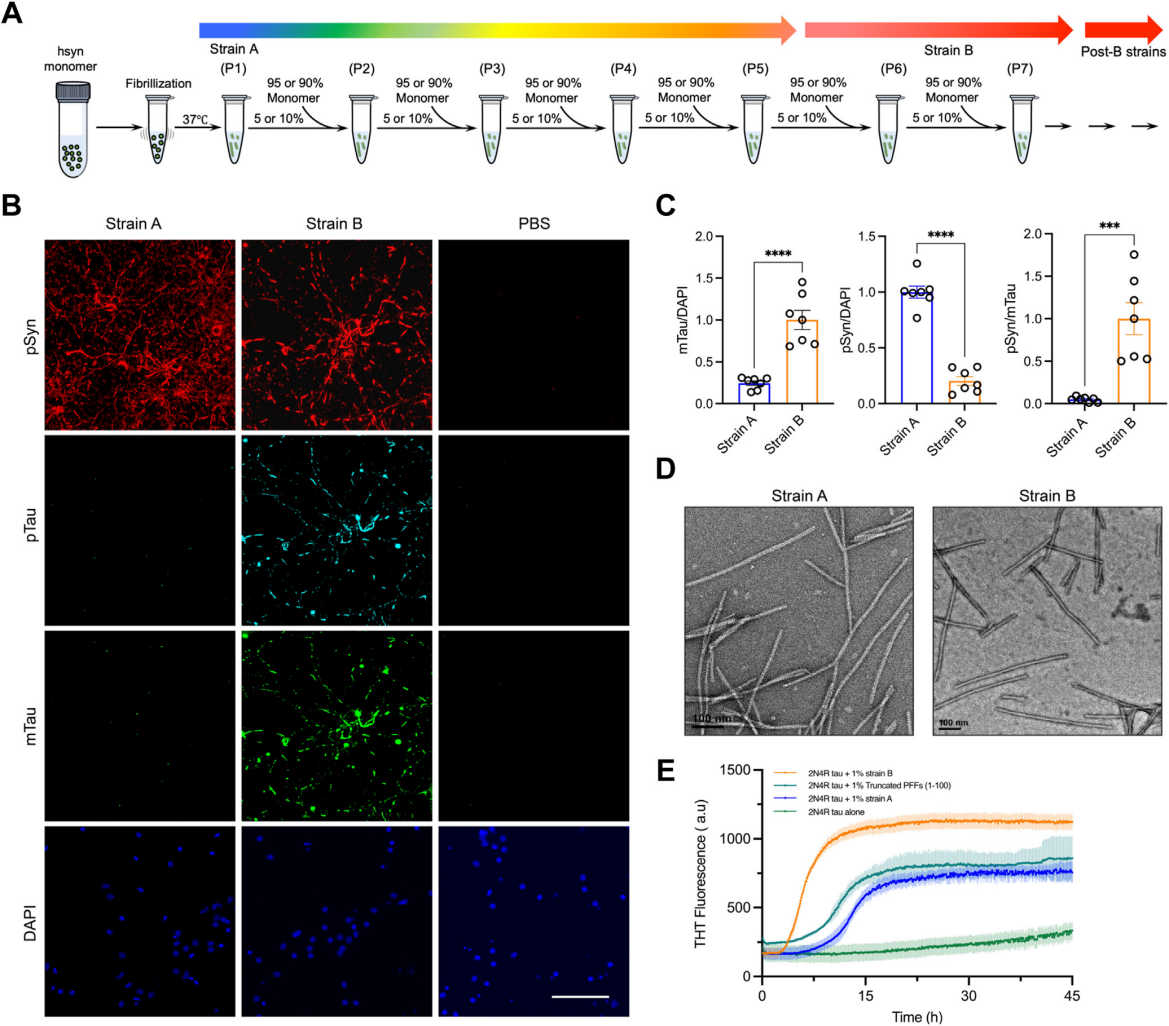
**The generation of α-syn strain B and its significant promotion of Tau inclusion in Neurons and *in vitro*.***A*, repetitive *in vitro* seeded fibrillization procedures lead to the transformation of full-length α-syn strain A into strains B and post-B strains. *B*, representative images of immunostaining for insoluble phospho-α-syn (pSyn), phospho-tau (pTau), and Mouse-Tau (mTau) after the addition of strain A or strain B α-syn into non-Tg neurons. 1% Triton X-100 was added to 4% PFA during fixing to remove soluble proteins. *C*, quantification of mTau immunostaining (*left*), phospho-α-syn (pSyn) immunostaining (*middle*), and pSyn *versus* total mTau shown in (*B*). *D*, negative Stain TEM images of *in vitro* formed α-syn strain A and strain B fibrils. Scalebar 100 nm. *E*, ThT assay for Wild-type Tau (2N4R) aggregation with and without different α-syn strains (*Orange*: 2N4R tau with 1% strain B; Deepteal: 2N4R tau with truncated PFFs (1–100); *Blue*: 2N4R tau with 1% strain A; *Green*: 2N4R tau alone).

In a previous study, Guo *et al.* identified two distinct strains of synthetic α-synuclein fibrils, each displaying significant differences in their efficiency to cross-seed tau aggregation in neuronal cultures and mice (23). strain A typically seeded aggregation of endogenous α-synuclein, while strain B, in contrast, seeded less α-synuclein but preferentially induced tau aggregation. The observed disparities in these outcomes sparked our curiosity about the differences between the two distinct strains. Consequently, to investigate their disparities, we initially employed identical methodologies to culture both strains, as illustrated in Figure 1*A*. strain A was the first generation of fibril produced through continuous oscillation, while strain B was obtained through successive seeding, spanning the sixth to seventh generations.

Subsequently, using Thioflavin T (THT) fluorescence assays, we separately incubated strain A and strain B with tau 2N4R protein. The fluorescence results indicated that strain B similarly demonstrated enhanced cross-seeding tau 2N4R capability compared to strain A, further substantiating its unique capacity to induce tau aggregation (Fig. 1*E*). This preference for strain B cross seeding of tau was also observed in cells, when we seeded primary neurons to evaluate the seeding capacities of the two strains. Our findings revealed that full-length strain B exhibited a superior ability to cross-seed tau protein compared to strain A (Fig. 1, *B* and *C*).

Negative staining TEM images of strain A and strain B were insufficient to distinguish them morphologically (Fig. 1*D*). However, we hypothesized that strain B would be structurally unique and chose to pursue its atomic structure. For instance, analyses conducted by Yang *et al.* and Manuel Schweighauser *et al.* on Multiple System Atrophy (MSA) and Dementia with Lewy Bodies (DLB) extracted amyloid fibrils from patient brain tissues, revealing different conformational isoforms. These investigations confirm that the polymorphic forms of α-synuclein exhibit differential seeding capacities. Therefore, resolving the high-resolution structure of strain B at the atomic level is of significant biological importance for understanding its pathological differences.

### CryoEM structure of **α**-syn strain B fibril polymorph

**Figure 2:**
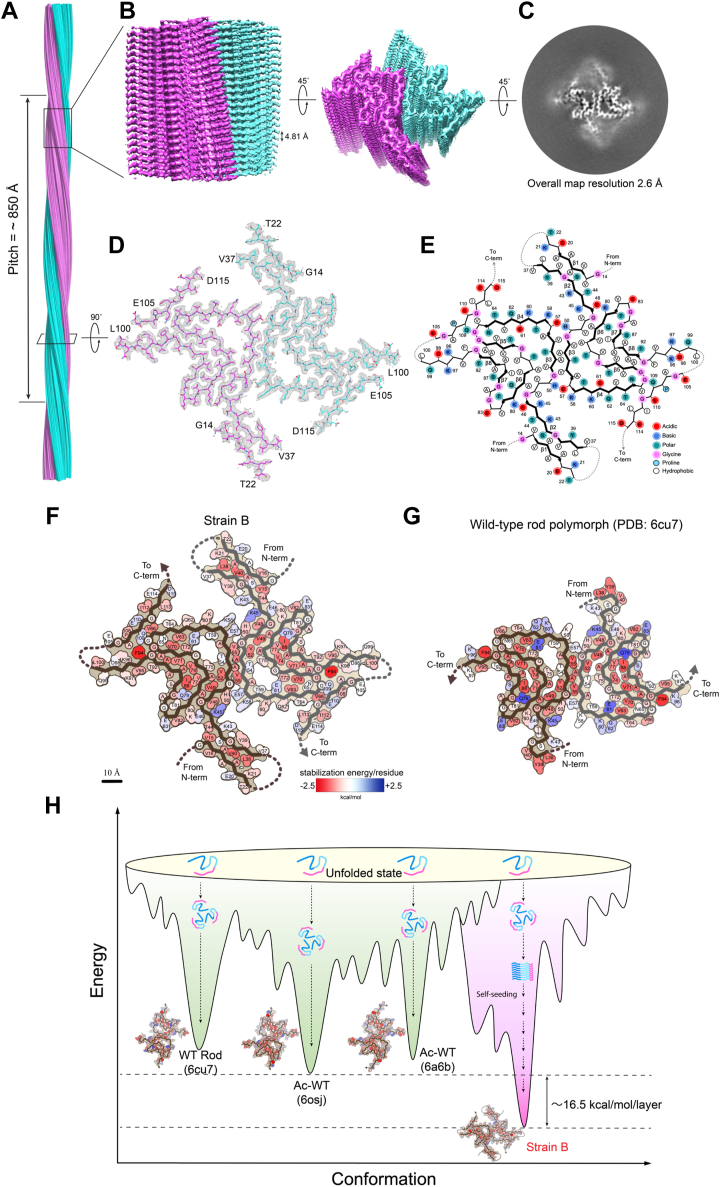
**CryoEM structure of α-syn strain B polymorphic fibril.***A* and *B*, cryoEM density map of the α-syn strain B fibril. Length of half pitch (180° helical turn), and helical rise is indicated. The two protofilaments are colored in *cyan* and *purple*. *C*, cross-sectional view of a density map projection. *D*, top view of strain B fibril. One layer of the structure is shown, which consists of two α-syn molecules covering residues 14 to 22…37 to 100…105 to 115. The two molecules are colored differently. *E*, schematic representation of one cross-sectional layer of the amyloid core, with β-strands shown as *thicker arrows* and the less ordered region (residues 22–37, 101–104) marked as *dotted lines*. *F* and *G*, schematic free energy of stabilization maps for strain B and strain A. *H*, the misfolding landscape indicates that strain B, cultured through a passage, exhibits a more organized and low-energy arrangement compared to previously identified polymorphs.

To understand the structural basis for the unique properties of strain B fibrils, we pursued their atomic structure using cryoEM. The strain B fibril samples were immobilized on a carbon grid and frozen in liquid ethane. After screening and optimizing the conditions for the frozen grid, cryo-electron micrographs were collected at a magnification of 81,000 times using a 300 keV Titan Krios microscope. 46,626 fibrils picked from 2934 micrographs were used for the reconstruction of strain B fibril (Table S1). The strain B fibril samples were morphologically homogeneous and showed a single dominant species during 2D classification (Fig. S3, *A* and *B*). After helical reconstruction of the main fibril species using Relion 3.1, we obtained a 3D density map of the strain B fibril with an overall resolution of 2.61 Å (Figs. 2, S2, and S3, *C* and *D*). The fibril contains two filaments intertwined along a principal helical axis (Fig. 2). The helical twist between α-syn subunits is −179.47° and the helical rise is 2.408 Å (Fig. 2*C* and Table S1). The density map indicates a left-handed helix with a pitch of about ∼85 nm (Fig. 2*A*).

**Figure 3:**
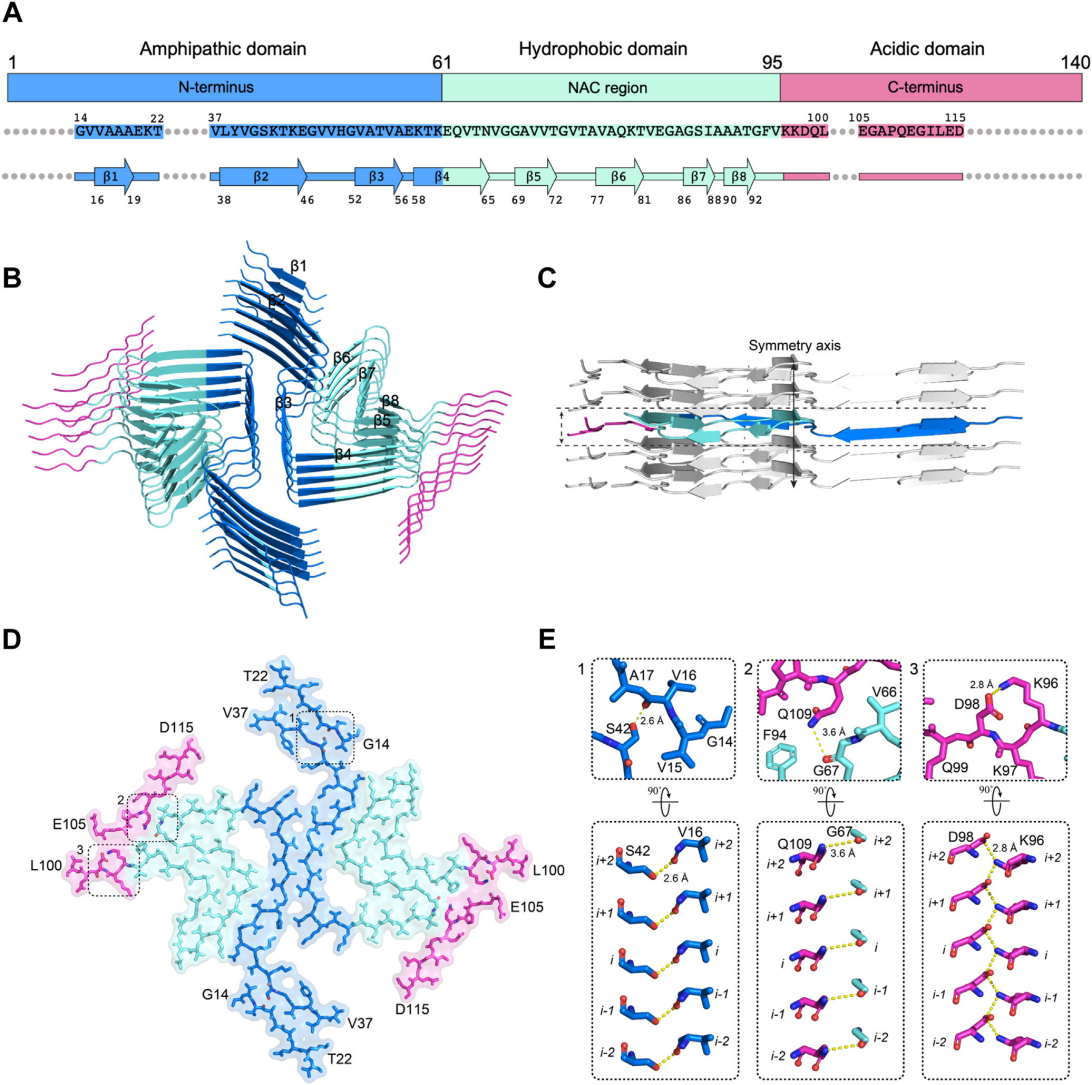
**Structure and interaction analysis of α-syn strain B.***A*, schematic of the primary and secondary structure of the fibril core of strain B protofilament. *Arrows* indicate regions of strain B that adopt β-strand conformations. N-terminus, NAC domain, and C-terminus are shown in *sky blue*, *pale cyan*, and *pink colors*, respectively. *B*, views of five layers of the strain B fibril are shown in the cartoon. The β strands are numbered and labeled. *C*, ribbon representation of five stacked layers of the fibril core shows the β-strand segments, with the distance between neighboring layers in one protofilament is indicated. *D*, one layer of strain B fibril is shown by *sticks*, with the *dashed boxes* labeled 1, 2, and three highlighting the critical interaction regions in both the N-terminus and C-terminus. *E*, magnified top views of the boxed regions (1–3) in (*D*) show the key H-bond and electrostatic interactions in strain B fibrils. In the N-terminus, Val16 and Ser42 form a hydrogen bond, and Gly67 and Gln109 form another hydrogen bond. In the C-terminus, Lys96 and Asp98 form a salt bridge. A side view highlights the critical interaction between two residues from opposing subunits, with distances of 2.6, 3.6, and 2.8 Å (*black*).

From the high-resolution atomic structure model of strain B fibril, we observed the classic Greek key topology within the fibril core (Figs. 2*E* and 3, *A*–*C*). This core comprised residues 37 to 100 of full-length α-synuclein (α-syn) protein, slightly larger than the wild-type (WT) α-syn fibrils (composed of residues 38–97). Notably, unlike the previously determined WT fibrils, the N-terminal and C-terminal regions of strain B fibrils are adsorbed onto and extend the fibril core. Density islands at both ends of the fibril core (Fig. S2, *A* and *B*) are sufficiently resolved to visualize their side-chain densities apart from the main chain. Consequently, the structure of strain B reveals the distinct structures of residues 14 to 22 (^14^GVVAAAEKT^22^, island 1) at the N-terminus and residues 105 to 115 (^105^EGAPQEGILED^115^, island 2) at the C-terminus, contributing to the elongated fibril structure of strain B (Fig. 2, *D* and *E*). Specifically, island 1 (β1) packs together with the core structure β2, while island two participates in the assembly of β4 and β8 (Figs. 2*E* and 3, *A* and *B*). Thus, the ordered sequence of strain B fibril that we report includes residues 14 to 22, 37 to 100, and 105 to 115, forming the entire core structure. It is the longest α-synuclein fibril structure reported so far and is distinct from other α-synuclein fibrils (Figs. S1 and S2).

The resolution of 2.61 Å allowed us to directly visualize the side-chain densities in the "island" regions, which were unambiguously assigned to the N-terminal residues (14, 15, 16, 17, 18, 19, 20, 21, 22) and C-terminal residues (105–115) based on their sequence-specific features (Fig. S2). The high level of order observed in these densities excludes the possibility of disordered or surface-bound monomeric α-synuclein, which typically lacks clear side-chain density. Furthermore, the single, continuous fibril core revealed by the density map strongly argues against the presence of secondary nucleation mechanisms or surface-adsorbed monomeric α-synuclein. Unlike the irregular and heterogeneous density distributions typically associated with secondary nucleation, the "island" regions exhibit highly stable and ordered sequence-specific densities that align seamlessly with the fibril core. Together, these observations support the hypothesis that the "island" densities are structural extensions of the fibril core, rather than independent entities.

### The protofilament of **α**-syn strain B exhibits a prominently negatively charged surface

Compared to other reported polymorphs of α-synuclein fibrils, the structure of strain B possesses a common, conserved β-arch fibril core and has an identical fibril zipper interface formed by residues 50 to 57 (^50^HGVATVAE^57^) from the preNAC region (Fig. 2, *E*–*G*). The main difference between strain B and others lies in the N-terminal and C-terminal regions of the fibril. In strain B, residues 14 to 22 of the N-terminus and 105 to 115 of the C-terminus also participate in the assembly of the fibril core structure (Fig. 2, *D* and *E*). Residues A17, A18, and A19 at the N-terminus form hydrophobic interactions with residues L38 and V40. Residues V16 and S42 form hydrogen bonds, stabilizing the β-sheet structure of residues 14 to 22 at the N-terminus. Additionally, residue Q109 at the C-terminus forms a hydrogen bond with G67, and the electrostatic interaction between K96 and D98 plays a stabilizing role, locking the flexibility of the C-terminal structure (Figs. 3*D* and *E* and S1).

Moreover, the charge distribution indicates a higher number of negatively charged residues on the fibril surface, attributed to the increased involvement of N-terminal and C-terminal flexible sequences in forming the fibril core. For example, the negatively charged side chains of residues E22, E105, E110, E114, and D115 are all solvent exposed (Fig. 2, *E* and *F*). This significant change in surface charge properties may lead to different pathological characteristics of amyloid fibrils. Furthermore, through the calculation of solvation energy, strain B, due to the participation of more residues, exhibits a lower energy state, suggesting a more stable fibril structure (Fig. 2*H*). Through *in vitro* SDS treatment experiments, we also found that strain B indeed shows more stability than strain A (Fig. S6).

### Inhibiting D105-E115 effectively reduces α-syn strain B-induced tau aggregation

We next sought to understand the participation of the N-terminal residues 14 to 22 and the C-terminal residues 105 to 115 in the strain B fibril core. We hypothesized that their negatively charged surface and the additional presence of a stretch of 14 negatively charged amino acids at the C-terminus, spanning residues 104 to 140, might complement the microtubule-binding domain of tau. That domain is enriched with positively charged amino acids, such as lysine and arginine, and is located at the tau C-terminus (Fig. S5) (24, 25, 26, 27, 28). This region also plays a crucial role in the regulation of tau protein phosphorylation, abnormalities of which have been implicated in the onset and progression of neurodegenerative diseases. Moreover, considerable evidence suggests a possible electrostatic interaction between the negatively charged residues at the C-terminus of α-synuclein and the positively charged amino acid residues of tau protein, facilitating the mutual association of these two proteins (29, 30).

**Figure 4:**
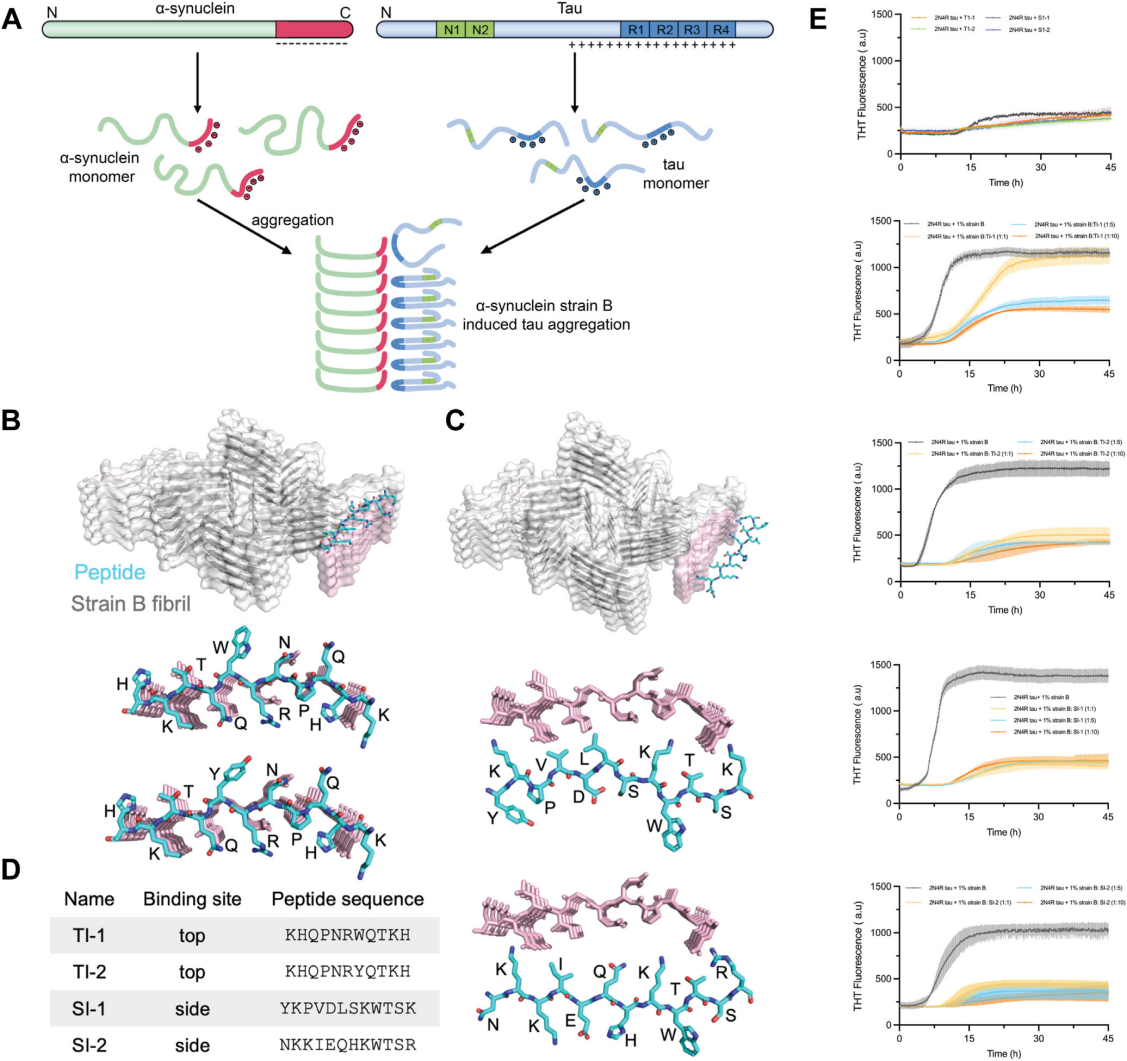
**The structured C-terminal domain of α-synuclein contributes to the development of Tau aggregates.***A*, schematic representation of α-synuclein fibrils promoting the aggregation of Tau protein. *B–D*, the design strategy of peptide inhibitors based on the C-terminal region of strain B fibril includes capping block and side block inhibitor design. *E*, thioflavin T assay to measure Tau aggregation and the effect of inhibitors. 50 μM Tau monomer was aggregated in the presence of 1% strain B and different inhibitors (Tau: inhibitor molar ratios, 1:1, 1:5, 1:10); All four inhibitors decreased ThT fluorescence indicative of inhibition of aggregation. Each curve is an average of three data sets where the error bars represent standard deviations.

Therefore, based on these findings, it is reasonable to hypothesize that negatively charged residues contributed by the D105-E115 segment of strain B fibrils may directly interact with tau protein, thereby promoting tau protein aggregation (Fig. 4*A*). To test this hypothesis, we designed two classes of peptide inhibitors based on the acquired three-dimensional structure of the D105-E115 segment of strain B. Using the Rosetta protein modeling suite, we systematically designed these peptides to optimize their binding affinity and specificity. The Rosetta FlexPepDock protocol was employed to predict peptide-protein interactions, enabling the identification of sequences that would effectively target the fibril structure. Energy minimization and docking simulations were performed to refine the binding conformation and enhance stability. One class (TI-1 and TI-2) targets the top of the fibril by binding to and capping the D105-E115 region, aiming to restrict the elongation of α-synuclein protofibrils (Fig. 4*B*). The second class (SI-1 and SI-2) targets the lateral side of the D105-E115 protofibrils to hinder the potential binding of α-synuclein protofibrils with tau protein (Fig. 4*C*). Subsequently, the synthesized peptides underwent *in vitro* thioflavin T (THT) fluorescence assays. The results of the *in vitro* Thioflavin T (THT) fluorescence assays demonstrated that both classes of peptides significantly reduced the ability of strain B cross-seeded tau aggregation (Figs. 4*E* and S9, *A*–*D*). The Thioflavin T (ThT) fluorescence assay results demonstrated that TI-1, TI-2, SI-1, and SI-2 all dose-dependently inhibit the formation of α-synuclein cross-seeded tau fibrils. Notably, treatment with higher concentrations of SI-1 and SI-2 reduced ThT fluorescence intensity by approximately 60%-70%, indicating that SI-1 and SI-2 exhibit a stronger inhibitory effect (Figs. 4*E* and S9). Moreover, our experimental results indicate that SI-1 can slightly promote the aggregation of 2N4R tau. Although the effect is not statistically significant, it is nonetheless observable. We speculate that this inhibitor may contain a higher proportion of hydrophobic amino acids compared to other inhibitors, which could influence the aggregation behavior of 2N4R tau. These findings underscore the critical role of synuclein residues D105-E115 in mediating strain B-induced tau aggregation, providing support for our proposed hypothesis.

## Discussion

The interaction between α-synuclein and tau proteins is a key area of research in neurodegenerative diseases, particularly in the context of Parkinson's disease (PD) and Alzheimer's disease (AD) (20, 31, 32, 33). These proteins are known for their tendency to misfold and aggregate, significantly contributing to pathological processes in these diseases (4, 18, 31, 34). α-synuclein is primarily located in presynaptic terminals and is involved in synaptic vesicle dynamics and neurotransmitter release, while tau protein is mainly found in the axonal compartment, where it stabilizes microtubules and maintains neuronal structure (18, 35, 36, 37). Pathological aggregation of α-synuclein and tau disrupts neuronal function, leading to diseases such as Parkinson's disease (PD) and Alzheimer's disease (AD), which are characterized by the presence of Lewy bodies and neurofibrillary tangles, respectively (4, 15, 38, 39).

Recent studies have highlighted the potential for these proteins to interact and influence each other's aggregation process, a phenomenon known as cross-seeding (40, 41, 42). The cross-seeding mechanism implies that misfolded forms of one protein can trigger the aggregation of another protein, worsening neurodegenerative pathways and resulting in the overlap observed in synucleinopathies and tauopathies (43). Understanding the cross-seeding interaction between α-synuclein and tau is crucial for developing therapeutic strategies to alleviate their pathological effects. Studies have shown that αS can induce the phosphorylation and aggregation of tau protein, thereby weakening the microtubule-stabilizing function of tau protein and promoting the occurrence of tauopathy. These interactions not only amplify the aggregation cascade but also provide potential targets for therapeutic intervention, emphasizing the necessity for a comprehensive understanding of their molecular mechanisms.

By cryoEM helical reconstruction, this study resolves, for the first time, the atomic structure of a novel fibril identified as strain B. These fibrils are characterized by more ordered N-terminal (14-22) and C-terminal (105-115) structures. This conformation involves the largest number of α-synuclein residues observed to date, participating in the formation of the fibril core structure. Structural analysis revealed that the C-terminal negatively charged amino acid sequence D105-E115 is involved in fibril core formation, resulting in a significantly negatively charged surface of the protofilament. This electrostatic property may confer unique pathological characteristics to strain B. Combined with numerous previous studies, we hypothesize that this characteristic results in a robust electrostatic interaction between the positively charged area of the C-terminal of strain B and tau protein, consequently initiating abnormal tau protein aggregation. Based on this hypothesis, we designed activity experiments using specific peptide inhibitors targeting the C-terminally negatively charged amino acid sequence D105-E115. The experimental results confirmed our hypothesis that the peptide inhibitor binds to the negatively charged region at the C-terminal of α-synuclein, effectively inhibiting its facilitation of tau protein aggregation. This also sheds light on the molecular mechanism through which α-synuclein fibrils trigger tau protein aggregation.

Our experimental evidence indeed demonstrates the importance of the negatively charged region of the C-terminus in promoting α-synuclein tau aggregation. However, this does not mean that other domains lack functionality. In fact, as demonstrated by Guo *et al.*, deletion of the N-terminal domain also results in a loss of α-synuclein cross-seeding ability (23). We speculate that this may be attributed to the potentially critical role of the N-terminal domain in initiating and maintaining the conformation of amyloid fibrils. Once the N-terminal domain is deleted, the fibril conformation changes, leading to the loss of cross-seeding function. Furthermore, from the amyloid fibril structures extracted from the brains of patients, we observe that, in addition to the core structure, there are N-terminal or undetermined polypeptide densities present in Parkinson's disease (PD) and juvenile-onset synucleinopathy fibrils (JOS) (Fig. S8) (44, 45). We hypothesize that these structures, besides contributing to fibril aggregation, may also influence the pathological properties of α-synuclein fibrils. However, the specific molecular mechanisms behind this phenomenon may require further in-depth study.

The cross-seeding interaction between α-synuclein and tau involves multiple molecular mechanisms, the classification of which can aid in understanding the pathology of neurodegenerative diseases (43, 46, 47). For example, direct physical interactions between α-synuclein and tau can lead to the formation of copolymers that act as seeds to promote aggregation (19, 47). Misfolded α-synuclein can template tau into pathological conformations, similar to prion propagation (22). Through kinase activation, it induces phosphorylation, thereby increasing the aggregation tendency of tau (11). Additionally, α-synuclein-induced phosphorylation can disrupt the microtubule-stabilizing function of tau, leading to multiple complex and unclarified molecular mechanisms, such as microtubule disassembly and further aggregation (48, 49).

Recent advances in the interaction between α-synuclein and tau proteins have opened new avenues for therapeutic strategies in neurodegenerative diseases (4). Increasing evidence suggests that α-synuclein plays a crucial role in the pathogenesis of Parkinson's disease (PD) and other synucleinopathies through its ability to form toxic intracellular aggregates. The potential of α-synuclein as a therapeutic target is enormous, but researchers must find a delicate balance of its physiological roles in neurons, including its involvement in neurotransmitter release and synaptic vesicle transport. The discovery of a cross-seeding mechanism, where the polymerization of one protein is catalyzed by another protein, has profound implications for therapeutic intervention. Interactions between tau and α-synuclein promote each other's fibrillation and solubility, suggesting that deleterious feed-forward loops are critical for the initiation and progression of neurodegeneration. Understanding these mechanisms may help in developing new therapeutic strategies aimed at blocking the spread of neurodegenerative fibrils and offer a newly identified target for new drugs.

## Experimental procedures

### Purification and *in vitro* fibrillization of recombinant **α**-synuclein

The full-length α-synuclein (FL α-syn, amino acids 1–140) was produced in BL21 (DE3) cells and C-terminally truncated α-syn (1–100) were produced in BL21 (DE3) cells and purified using the method outlined by Li *et al.* and Ni X *et al.* (50, 51). Fibrillization was initiated by diluting the recombinant α-synuclein to a concentration of 5 mg/ml in Dulbecco's phosphate-buffered saline (PBS) with a pH of 7.0. The mixture was then incubated at 37 °C with continuous agitation at 1000 rpm for 5 to 7 days. The successful formation of fibrils was confirmed through negative staining electron microscopy (EM) and Thioflavin-T (ThT) binding assays. The resulting prefibrillar fibrils were stored at room temperature to prevent freeze-thaw cycles.

For repetitive self-seeding, 5% or 10% of the existing fibrils were added to fresh monomers to initiate subsequent passages until reaching the 10th passage (P10). When fibrillization was seeded by strains A or B fibrils, 10% of the existing fibrils were included in the reaction. All seeded fibrillization procedures followed the same conditions as the *de novo* fibrillization process. Each α-synuclein construct was tested using at least two independently prepared monomer batches where each monomer batch underwent at least two series of self-seeded fibrillization experiments.

### Expression and purification recombinant tau (2N4R)

Expression of tau was carried out in *E. coli* BL21 (DE3) competent cells, as described by Zhang (2020). In brief, protein expression was induced at an OD600 of 0.8 with 1 mM isopropyl β-D-thiogalactopyranoside (IPTG) for 15 h at 25 °C. Cells were harvested by centrifugation (4000 rpm for 10 min at 4 °C) and resuspended in lysis buffer (20 mM Tris-HCl at pH 7.5, 150 mM NaCl, 1 mM EDTA, 2 mM DTT, supplemented with 0.1 mM PMSF and 1 × EDTA-free protease cocktail inhibitors). Cell lysis was performed using sonication at 60% amplitude with a Sonics VCX-750 Vibracell Ultrasonic Processor for 10 min, 3 s on/3 s off). Lysed cells were centrifuged at 15,000 × rpm for 60 min at 4 °C, filtered through 0.22 μm cut-off filters, and loaded onto a HiTrap SP HP 5 ml column (GE Healthcare) for cation exchange. The column was washed with 10 column volumes of lysis buffer and eluted using a gradient of wash buffer containing 0 to 1 M NaCl. Fractions of 3.5 ml were collected and analyzed by SDS-PAGE using either Bis-Tris 4 to 12% or Tris-Glycine 4 to 20%. Protein-containing fractions were pooled and precipitated using 0.35 g/ml of ammonium sulfate and left on a rocker for 30 min at 4 °C. Precipitated proteins were then centrifuged at 15,000 × rpm for 20 min at 4 °C and resuspended in 10 ml of 20 mM Tris-HCl, pH 7.5, 150 mM NaCl with 5 mM DTT, and loaded onto a Superdex 200 Increase 10/300 size exclusion column. Size exclusion fractions were analyzed by SDS-PAGE. Protein-containing fractions were pooled and concentrated to 8 mg/ml using molecular weight concentrators with a cut-off filter. Purified protein samples were flash-frozen in 100 μl aliquots for future use.

### Primary neuron cultures and fibril transduction

Primary mouse neurons were dissected on embryonic day 16 to 19 as described in a previous paper (1). Neurons were plated at a density of 100,000 cells per well in 24-well plates with coverslips. α-synuclein strain A and strain B fibrils were sonicated for 20 cycles (30 s on, 30 s off) using QSonica Microson XL-2000. Sonicated fibrils were then added on day 10 *in vitro*. Neurons were fixed and stained on day 14 post-transduction with MAP2 (#822501, Biolegend), AT8 (#MN1020, ThermoFisher scientific), 81A (#825701, Biolegend), and DAPI (D1306, Invitrogen). Images were taken with a Nikon microscope at 10 × magnification. Three fields were randomly selected for each data point. Area fractions were calculated using ImageJ.

### Immunocytochemistry and quantification

Neurons were collected for immunocytochemistry at 14 days post-treatment. Cells were washed with DPBS then fixed with 4% PFA (PFA; Electron Microscopy Sciences) containing 1% Triton X-100 for 15 min to remove soluble proteins. Following 1 h in 3% BSA and 3% FBS in DPBS blocking buffer at room temperature, neurons were incubated with anti-phospho-α-syn (81A) and anti-neurofilament antibody (NFL) overnight at 4 °C followed by staining with secondary antibodies for 2 h at room temperature. The plate was scanned on an ImageXpress Pico system scanner. Quantification of the area occupied by α-syn fibrils and tau induced pathology was performed by Cell Reporter Xpress.

### ThT kinetic assay

All the purified α-syn monomers (50 μM) and full-length tau(2N4R) monomers were adequately mixed with 10 μM ThT and added into a 96-well-plate (final volume of 100 μl). Samples were incubated at 37  °C for over 3 days with 600 rpm double orbital shaking. The ThT signal was monitored using the FLUOstar Omega Microplate Reader (BMG Labtech) at an excitation wavelength of 440 nm and an emission wavelength of 490 nm.

### SDS stability assay

The SDS stability of stain A and strain B α-syn fibrils were evaluated using the aggregation tendency after the treatment of SDS with different concentrations (0.5% ∼ 3.5%). The ThT signals were measured to monitor the aggregation of stain A and strain B α-syn after 5 min of SDS treatments at 37 °C with double orbital shaking at 600 rpm. The 10% SDS was added to reach different SDS final concentrations ranging from 0.5% to 3.5%. The ThT signals with 0% SDS treatment were used for normalization.

### Negative stain transmission electron microscopy (TEM)

An aliquot of 2.5 μl fibril sample was spotted onto a freshly glow-discharged carbon-coated electron microscopy grid for 2 min, then 5 μl uranyl acetate (2% in aqueous solution) was applied to the grid for 1 min. The excess stain was removed by filter paper. Another 3 μl uranyl acetate was applied to the grid and then immediately removed. The samples were imaged using an FEI T12 electron microscope.

### Structure-based design of peptide inhibitors

Computational designs were performed by adopting the Design protocol in Rosetta version 3.8, following the methods previously described (52). Briefly, the structure of the prefibrillar fibril strain B was used as a design template. An extended L-amino acid blocker peptide is aligned with the fibril structure, on the top and side of the β-sheet. Full sequence optimization of the extended peptide was performed, and rigid body orientation, backbone conformation, and side-chain packing of the blocking peptide were optimized for each sequence. Shape complementarity and buried surface area were calculated for each design. The designs were ranked by total binding energy, and the top-ranking peptides were synthesized by GenScript and further tested.

### Quantification of fibrillar species

The separation and quantification of fibrils were carried out as described previously (53). A 50 μM monomeric Tau solution, supplemented with different inhibitors, was incubated in a 96-well plate at 37 °C with 500 rpm shaking in the presence of Teflon polybeads for 3 days. Fibrils formed during incubation were pelleted by ultracentrifugation using an Optima MAX-TL centrifuge equipped with a TLA120.2 rotor at 60,000 rpm for 1.5 h at 4 °C. The supernatant and pellet fractions were collected separately, and the fibrillar Tau content was quantified by subtracting the protein concentration in the supernatant from that in the pellet fraction.

### CryoEM sample preparation, movie acquisition, and drift correction

An aliquot of 2.5 μl of fibril solution was applied to a Quantifoil holey carbon grid (2/1, 300 mesh), that was glow discharged for 40 s with a PELCO Easiglow system. The grid was blotted and plunge-frozen in liquid ethane with a Vitrobot IV (Thermo Fisher) at 4 °C under 100% humidity. The frozen grids were stored in liquid nitrogen before use. For data collection, the cryoEM grids were loaded into an FEI Titan Krios electron microscope equipped with Gatan BioContinuum GIF and K3 direct detector. The Movies were recorded as dose-fractionated frames in super-resolution mode by the SerialEM automation software package, with image shift induced-beam tilt correction (54). The slit width in the GIF system was set to 20 eV to remove inelastically scattered electrons. A total of 4095 movies were recorded for the data set at a nominal magnification of ×81,000, corresponding to a calibrated pixel size of 0.5395 Å on the specimen. An exposure time of 3.6 s was used at a rate of 0.055 s per frame and the dose rate per frame was set to 0.74 e−/Å^2^, producing 65 frames and a total dosage of 48 e−/Å^2^.

Frames in each movie were aligned for drift correction with the graphics processing unit (GPU)-accelerated program MotionCor2 (55). The first and last frames were discarded during drift correction. Two averaged micrographs, one with dose weighting and another without dose weighting, were generated for each movie after drift correction. The averaged micrographs were binned 2 × 2 to yield a pixel size of 1.079 Å. The micrographs without dose weighting were used for CTF estimation and particle picking, while those with dose weighting were used for particle extraction and in-depth processing.

### CryoEM data processing

The CTF estimation of each micrograph was performed by CTFFIND4 (56). By discarding the micrographs either with underfocus values outside the allowed range (1.0–3.0 μm), or containing crystalline ice, we selected 2934 good ones from the dataset. The filaments were preliminarily picked using crYOLO (57, 58) and the manual inspection of filament picking was performed subsequently. A total of 57,074 Fibril particles were first extracted using a large box size (1024 pixels) and a 10% inter-box distance in Relion v.3.1 (59), then the particles were subjected to two-dimensional (2D) class averaging to determine the pitch. Helical parameters were deduced from the pitch with the assumption that each helix had a twisted twofold screw axis. The 2D classes reveal that strain B α-syn forms fibrils of a single morphology with a pitch of ∼850 Å (Fig. S3*B*), given the calculated helical twist of ∼179.49° (helical rise of ∼2.4 Å). Subsequently, we extracted all fibril particles with a 512-pixel box size and 10% inter-box distance, yielding 170,513 particles. Using the calculated helical twist (179.49°) and helical rise (2.4 Å), these particles were subjected to a three-dimensional (3D) class averaging with a single class and a featureless cylinder created by EMAN2 (60) as the initial model. The cylinder was refined to a model in which two separated and twisted protofilaments could be seen. This model was then used to classify good and bad particles with a 3D class averaging with three classes. Particles in the best class of the previous 3D classification were re-extracted with a 300-pixel box size for further 3D classification. One additional 3D classification was performed, and a final subset of 26,051 helical segments was selected and subjected to 3D auto-refinement, CTF refinement, and post-processing, yielding a map at 2.61 Å resolution (Fig. S3). Details of the data processing are summarized in Table S1.

The global resolution reported above is based on the “gold standard” refinement procedures and the 0.143 Fourier shell correlation (FSC) criterion. Local resolution evaluation was performed with Relion v.3.1 (Fig. S3).

### Atomic model building

Atomic model building was accomplished in an iterative process involving Chimera, Coot, and Phenix (61, 62, 63). Briefly, the structure of Rod polymorph α-syn (PDB: 6CU7) was fitted into the cryoEM map as an initial model by Chimera (50). This fit revealed the mismatched segments of the mainchain. After deleting the mismatched region, the model was refined by “real-space refinement” in Phenix. We then manually built the missing residues and adjusted side chains to match the cryoEM map with Coot. This process of real space refinement and manual adjustment steps was repeated iteratively until the peptide backbone and side chain conformations were optimized. Ramachandran, secondary-structure restraints, and NCS restraints were used during the refinement. Refinement statistics are summarized in Table S1. The model was also evaluated based on Ramachandran plots and MolProbity scores in Table S1 (64).

## Data availability

Atomic coordinates have been deposited in the Protein Data Bank under accession number 9C5R. The cryoEM density map has been deposited in the Electron Microscopy Data Bank under accession number EMD-45221.

## Supporting information

This article contains supporting information.

## Conflicts of interests

The authors declare that they have no conflicts of interest with the contents of this article.

## References

[1] Lee V.M., Goedert M., Trojanowski J.Q. (2001). Neurodegenerative tauopathies. Annu. Rev. Neurosci..

[2] Dugger B.N., Dickson D.W. (2017). Pathology of neurodegenerative diseases. Cold Spring Harb. Perspect. Biol..

[3] Irwin D.J., Hurtig H.I. (2018). The contribution of tau, amyloid-beta and alpha-synuclein pathology to dementia in Lewy body disorders. J. Alzheimers Dis. Parkinsonism.

[4] Vasili E., Dominguez-Meijide A., Outeiro T.F. (2019). Spreading of alpha-synuclein and tau: a systematic comparison of the mechanisms involved. Front. Mol. Neurosci..

[5] Bonini N.M., Giasson B.I. (2005). Snaring the function of alpha-synuclein. Cell.

[6] Chandra S., Gallardo G., Fernandez-Chacon R., Schluter O.M., Sudhof T.C. (2005). Alpha-synuclein cooperates with CSPalpha in preventing neurodegeneration. Cell.

[7] Bartels T., Choi J.G., Selkoe D.J. (2011). alpha-Synuclein occurs physiologically as a helically folded tetramer that resists aggregation. Nature.

[8] Goedert M., Spillantini M.G., Serpell L.C., Berriman J., Smith M.J., Jakes R. (2001). From genetics to pathology: tau and alpha-synuclein assemblies in neurodegenerative diseases. Philos. Trans. R. Soc. Lond. B Biol. Sci..

[9] Goedert M. (2001). The significance of tau and alpha-synuclein inclusions in neurodegenerative diseases. Curr. Opin. Genet. Dev..

[10] Giasson B.I., Forman M.S., Higuchi M., Golbe L.I., Graves C.L., Kotzbauer P.T. (2003). Initiation and synergistic fibrillization of tau and alpha-synuclein. Science.

[11] Irwin D.J., Lee V.M., Trojanowski J.Q. (2013). Parkinson's disease dementia: convergence of alpha-synuclein, tau and amyloid-beta pathologies. Nat. Rev. Neurosci..

[12] Grundke-Iqbal I., Iqbal K., Tung Y.C., Quinlan M., Wisniewski H.M., Binder L.I. (1986). Abnormal phosphorylation of the microtubule-associated protein tau (tau) in Alzheimer cytoskeletal pathology. Proc. Natl. Acad. Sci. U. S. A..

[13] Duan P., Dregni A.J., Mammeri N.E., Hong M. (2023). Structure of the nonhelical filament of the Alzheimer's disease tau core. Proc. Natl. Acad. Sci. U. S. A..

[14] Gong C.X., Liu F., Grundke-Iqbal I., Iqbal K. (2005). Post-translational modifications of tau protein in Alzheimer's disease. J. Neural Transm. (Vienna).

[15] Martin L., Latypova X., Terro F. (2011). Post-translational modifications of tau protein: implications for Alzheimer's disease. Neurochem. Int..

[16] Kyalu Ngoie Zola N., Balty C., Pyr Dit Ruys S., Vanparys A.A.T., Huyghe N.D.G., Herinckx G. (2023). Specific post-translational modifications of soluble tau protein distinguishes Alzheimer's disease and primary tauopathies. Nat. Commun..

[17] Fujishiro H., Tsuboi Y., Lin W.L., Uchikado H., Dickson D.W. (2008). Co-localization of tau and alpha-synuclein in the olfactory bulb in Alzheimer's disease with amygdala Lewy bodies. Acta Neuropathol..

[18] Jellinger K.A. (2011). Interaction between alpha-synuclein and other proteins in neurodegenerative disorders. ScientificWorldJournal.

[19] Li X., James S., Lei P. (2016). Interactions between alpha-synuclein and tau protein: implications to neurodegenerative disorders. J. Mol. Neurosci..

[20] Ishizawa T., Mattila P., Davies P., Wang D., Dickson D.W. (2003). Colocalization of tau and alpha-synuclein epitopes in Lewy bodies. J. Neuropathol. Exp. Neurol..

[21] Hudak A., Kusz E., Domonkos I., Josvay K., Kodamullil A.T., Szilak L. (2019). Contribution of syndecans to cellular uptake and fibrillation of alpha-synuclein and tau. Sci. Rep..

[22] Nonaka T., Watanabe S.T., Iwatsubo T., Hasegawa M. (2010). Seeded aggregation and toxicity of alpha-synuclein and tau: cellular models of neurodegenerative diseases. J. Biol. Chem..

[23] Guo J.L., Covell D.J., Daniels J.P., Iba M., Stieber A., Zhang B. (2013). Distinct alpha-synuclein strains differentially promote tau inclusions in neurons. Cell.

[24] Fitzpatrick A.W.P., Falcon B., He S., Murzin A.G., Murshudov G., Garringer H.J. (2017). Cryo-EM structures of tau filaments from Alzheimer's disease. Nature.

[25] Lovestam S., Koh F.A., van Knippenberg B., Kotecha A., Murzin A.G., Goedert M. (2022). Assembly of recombinant tau into filaments identical to those of Alzheimer's disease and chronic traumatic encephalopathy. Elife.

[26] Falcon B., Zhang W., Murzin A.G., Murshudov G., Garringer H.J., Vidal R. (2018). Structures of filaments from Pick's disease reveal a novel tau protein fold. Nature.

[27] Zhang W., Tarutani A., Newell K.L., Murzin A.G., Matsubara T., Falcon B. (2020). Novel tau filament fold in corticobasal degeneration. Nature.

[28] Shi Y., Zhang W., Yang Y., Murzin A.G., Falcon B., Kotecha A. (2021). Structure-based classification of tauopathies. Nature.

[29] Lu J., Zhang S., Ma X., Jia C., Liu Z., Huang C. (2020). Structural basis of the interplay between alpha-synuclein and Tau in regulating pathological amyloid aggregation. J. Biol. Chem..

[30] Dasari A.K.R., Kayed R., Wi S., Lim K.H. (2019). Tau interacts with the C-terminal region of alpha-synuclein, promoting formation of toxic aggregates with distinct molecular conformations. Biochemistry.

[31] Yan X., Uronen R.L., Huttunen H.J. (2020). The interaction of alpha-synuclein and Tau: a molecular conspiracy in neurodegeneration?. Semin. Cell Dev. Biol..

[32] Sahni N., Yi S., Taipale M., Fuxman Bass J.I., Coulombe-Huntington J., Yang F. (2015). Widespread macromolecular interaction perturbations in human genetic disorders. Cell.

[33] Fahn S. (2003). Description of Parkinson's disease as a clinical syndrome. Ann. N. Y Acad. Sci..

[34] Negi S., Khurana N., Duggal N. (2024). The misfolding mystery: alpha-syn and the pathogenesis of Parkinson's disease. Neurochem. Int..

[35] Poewe W., Seppi K., Tanner C.M., Halliday G.M., Brundin P., Volkmann J. (2017). Parkinson disease. Nat. Rev. Dis. Primers.

[36] Scheres S.H.W., Ryskeldi-Falcon B., Goedert M. (2023). Molecular pathology of neurodegenerative diseases by cryo-EM of amyloids. Nature.

[37] Fu J., Gao X., Lu Y., Lu F., Wang Y., Chen P. (2024). Integrated proteomics and metabolomics reveals metabolism disorders in the alpha-syn mice and potential therapeutic effect of Acanthopanax senticosus extracts. J. Ethnopharmacol..

[38] Knopman D.S., Amieva H., Petersen R.C., Chetelat G., Holtzman D.M., Hyman B.T. (2021). Alzheimer disease. Nat. Rev. Dis. Primers.

[39] Martinez-Vicente M. (2012). Multiple ways for a-synuclein degradation. Mov Disord..

[40] Katorcha E., Makarava N., Lee Y.J., Lindberg I., Monteiro M.J., Kovacs G.G. (2017). Cross-seeding of prions by aggregated alpha-synuclein leads to transmissible spongiform encephalopathy. PLoS Pathog..

[41] Stoyka L.E., Mahoney C.L., Thrasher D.R., Russell D.L., Cook A.K., Harris A.T. (2021). Templated alpha-synuclein inclusion formation is independent of endogenous tau. eNeuro.

[42] Sengupta U., Puangmalai N., Bhatt N., Garcia S., Zhao Y., Kayed R. (2020). Polymorphic alpha-synuclein strains modified by dopamine and docosahexaenoic acid interact differentially with tau protein. Mol. Neurobiol..

[43] Lim K.H. (2019). Diverse misfolded conformational strains and cross-seeding of misfolded proteins implicated in neurodegenerative diseases. Front. Mol. Neurosci..

[44] Yang Y., Shi Y., Schweighauser M., Zhang X., Kotecha A., Murzin A.G. (2022). Structures of alpha-synuclein filaments from human brains with Lewy pathology. Nature.

[45] Yang Y., Garringer H.J., Shi Y., Lovestam S., Peak-Chew S., Zhang X. (2023). New SNCA mutation and structures of alpha-synuclein filaments from juvenile-onset synucleinopathy. Acta Neuropathol..

[46] Waxman E.A., Giasson B.I. (2011). Induction of intracellular tau aggregation is promoted by alpha-synuclein seeds and provides novel insights into the hyperphosphorylation of tau. J. Neurosci..

[47] Williams T., Sorrentino Z., Weinrich M., Giasson B.I., Chakrabarty P. (2020). Differential cross-seeding properties of tau and alpha-synuclein in mouse models of tauopathy and synucleinopathy. Brain Commun..

[48] Moussaud S., Jones D.R., Moussaud-Lamodiere E.L., Delenclos M., Ross O.A., McLean P.J. (2014). Alpha-synuclein and tau: teammates in neurodegeneration?. Mol. Neurodegener.

[49] Falcon B., Cavallini A., Angers R., Glover S., Murray T.K., Barnham L. (2015). Conformation determines the seeding potencies of native and recombinant Tau aggregates. J. Biol. Chem..

[50] Li B., Ge P., Murray K.A., Sheth P., Zhang M., Nair G. (2018). Cryo-EM of full-length alpha-synuclein reveals fibril polymorphs with a common structural kernel. Nat. Commun..

[51] Ni X., McGlinchey R.P., Jiang J., Lee J.C. (2019). Structural insights into alpha-synuclein fibril polymorphism: effects of Parkinson's disease-related C-terminal truncations. J. Mol. Biol..

[52] Sievers S.A., Karanicolas J., Chang H.W., Zhao A., Jiang L., Zirafi O. (2011). Structure-based design of non-natural amino-acid inhibitors of amyloid fibril formation. Nature.

[53] Kumar S.T., Donzelli S., Chiki A., Syed M.M.K., Lashuel H.A. (2020). A simple, versatile and robust centrifugation-based filtration protocol for the isolation and quantification of alpha-synuclein monomers, oligomers and fibrils: towards improving experimental reproducibility in alpha-synuclein research. J. Neurochem..

[54] Mastronarde D.N. (2005). Automated electron microscope tomography using robust prediction of specimen movements. J. Struct. Biol..

[55] Zheng S.Q., Palovcak E., Armache J.P., Verba K.A., Cheng Y., Agard D.A. (2017). MotionCor2: anisotropic correction of beam-induced motion for improved cryo-electron microscopy. Nat. Methods.

[56] Rohou A., Grigorieff N. (2015). CTFFIND4: fast and accurate defocus estimation from electron micrographs. J. Struct. Biol..

[57] Wagner T., Merino F., Stabrin M., Moriya T., Antoni C., Apelbaum A. (2019). SPHIRE-crYOLO is a fast and accurate fully automated particle picker for cryo-EM. Commun. Biol..

[58] Wagner T., Lusnig L., Pospich S., Stabrin M., Schonfeld F., Raunser S. (2020). Two particle-picking procedures for filamentous proteins: SPHIRE-crYOLO filament mode and SPHIRE-STRIPER. Acta Crystallogr. D Struct. Biol..

[59] Scheres S.H. (2012). RELION: implementation of a Bayesian approach to cryo-EM structure determination. J. Struct. Biol..

[60] Tang G., Peng L., Baldwin P.R., Mann D.S., Jiang W., Rees I. (2007). EMAN2: an extensible image processing suite for electron microscopy. J. Struct. Biol..

[61] Emsley P., Lohkamp B., Scott W.G., Cowtan K. (2010). Features and development of Coot. Acta Crystallogr. D Biol. Crystallogr..

[62] Adams P.D., Afonine P.V., Bunkoczi G., Chen V.B., Davis I.W., Echols N. (2010). PHENIX: a comprehensive Python-based system for macromolecular structure solution. Acta Crystallogr. D Biol. Crystallogr..

[63] Pettersen E.F., Goddard T.D., Huang C.C., Couch G.S., Greenblatt D.M., Meng E.C. (2004). UCSF Chimera--a visualization system for exploratory research and analysis. J. Comput. Chem..

[64] Chen V.B., Arendall W.B., Headd J.J., Keedy D.A., Immormino R.M., Kapral G.J. (2010). MolProbity: all-atom structure validation for macromolecular crystallography. Acta Crystallogr. D Biol. Crystallogr..

