# Supplementary material for: Structural basis of a distinct α-synuclein strain that promotes tau inclusion in neurons
Source: J Biol Chem. 2025 Feb 25;301(4):108351. doi: 10.1016/j.jbc.2025.108351 (PMC11982472; doi:10.1016/j.jbc.2025.108351)
Supplement: Figure S1 [file mmc1.pdf]

Figure S1

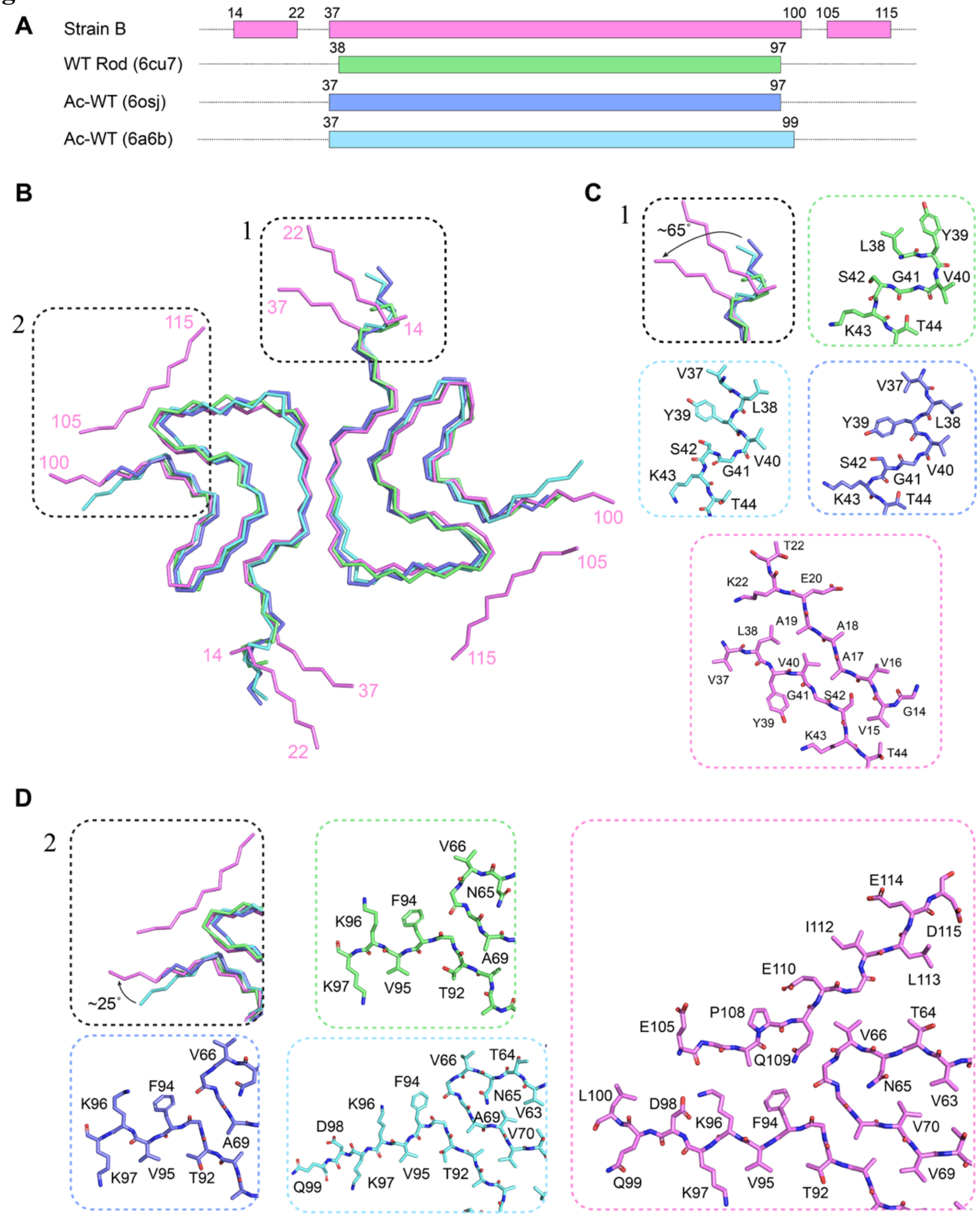

Figure S1. Structural comparison of different recombinant a-syn fibrils. (A) Schematic of the primary structure of the fibril core of strain B and other protofilaments. **(B-D)** Structure alignment of strain B with other recombinant protofilaments. Our α-Syn structure is purple in both overlays. Other structures are represented by green, blue, and cyan colors respectively. Boxes 1 and 2 show the differences between the N- and C-terminal domains of Strain B and other structures. Zoom-in images show specific details of the differences.
