# Supplementary material for: Structural basis of a distinct α-synuclein strain that promotes tau inclusion in neurons
Source: J Biol Chem. 2025 Feb 25;301(4):108351. doi: 10.1016/j.jbc.2025.108351 (PMC11982472; doi:10.1016/j.jbc.2025.108351)
Supplement: Figure S2 [file mmc2.pdf]

Figure S2

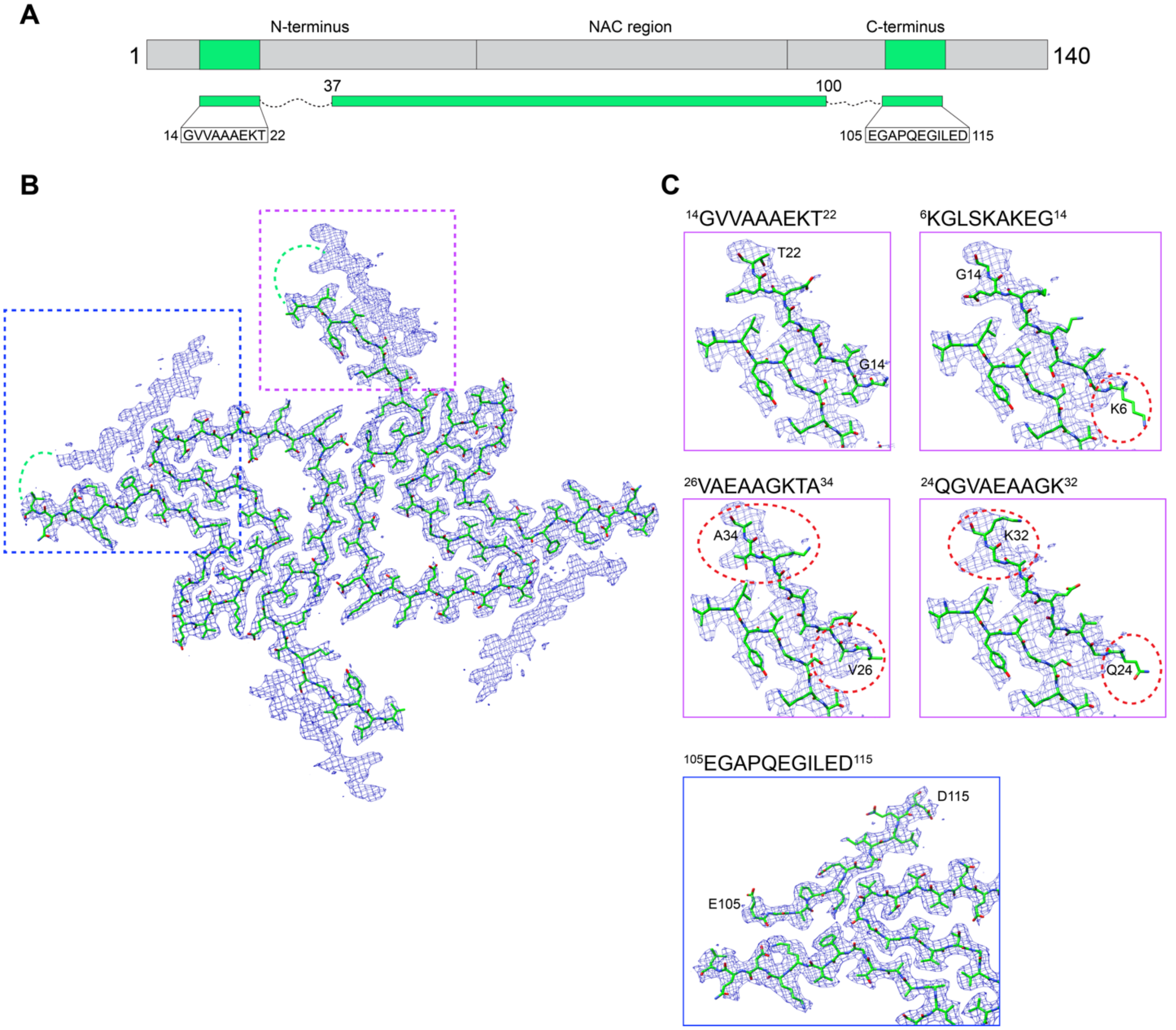

Figure S2. Speculative Atomic Model for Island suggested by the extra densities of strain B. (**A**) Schematic diagram shows the core sequence of strain B fibril (^{37}Val-^{100}Leu) and the island sequence (^{14}GVVAAAEKT^{22}) and (^{105}EGAPQEGILED^{115}). (**B, C**) Illustration of possible regions from strain B protofilament fibril that could occupy the 'Island'. Some residues from the N-terminal and C-terminal protofilament can give a reasonable explanation to the Island.
