# Supplementary material for: Structural basis of a distinct α-synuclein strain that promotes tau inclusion in neurons
Source: J Biol Chem. 2025 Feb 25;301(4):108351. doi: 10.1016/j.jbc.2025.108351 (PMC11982472; doi:10.1016/j.jbc.2025.108351)
Supplement: Figure S3 [file mmc3.pdf]

Figure S3

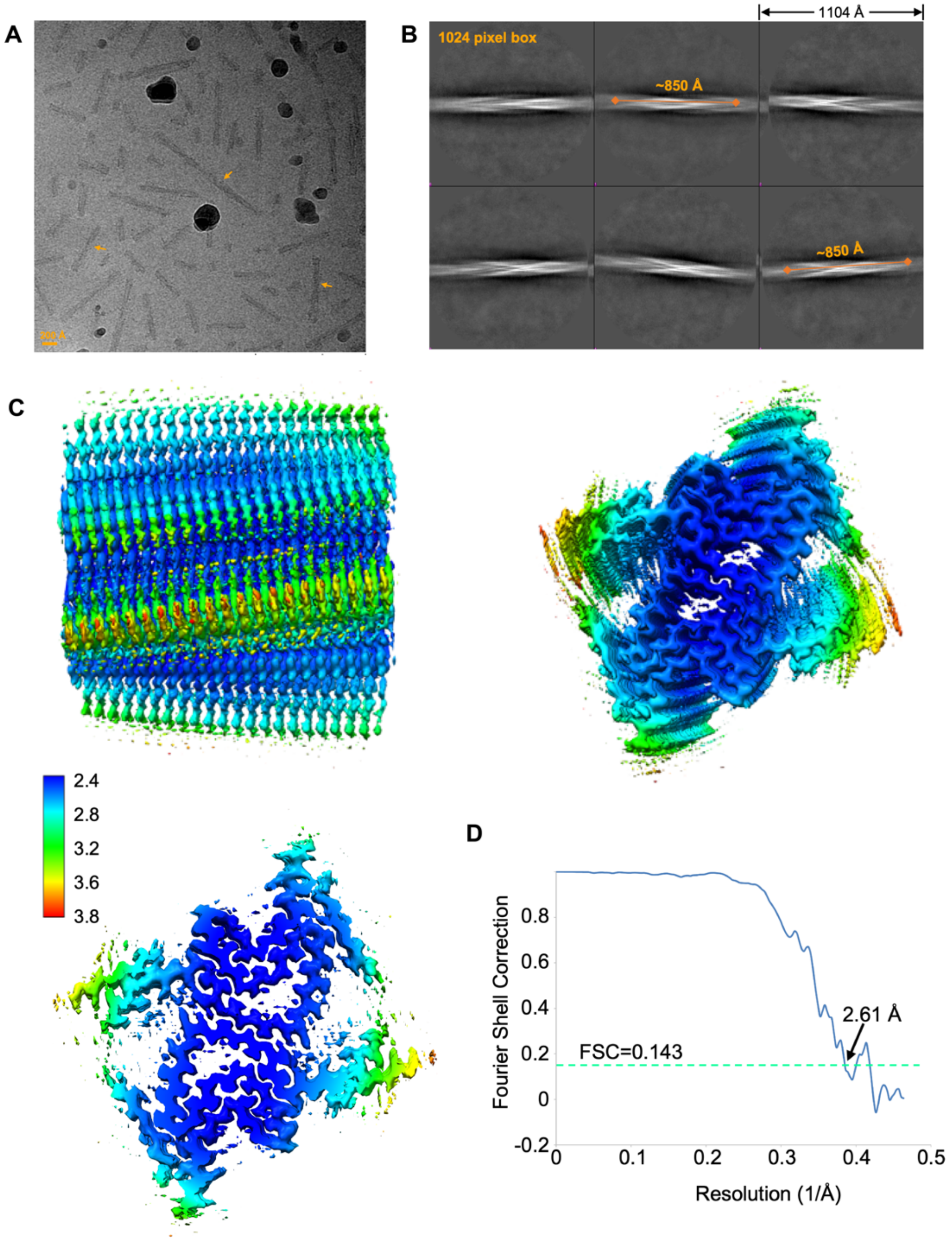

Figure S3. Resolution estimation of the cryo-EM map of strain B α-syn fibril. (A) A representative cryo-EM micrograph. Scale bar, 200 Å. **(B)** Representative 2D class averages at the 1024-pixel box (1104 Å box length). Several classes show that the crossover distance of the fibril is about 850 Å. **(C)** Local resolution estimation. Density maps are colored based on the local resolution; the resolution of the core region is around 2.61 Å. The density map of strain B fibrils is colored according to local resolution estimated by ResMap. The enlarged cross sections and top view show the density map of two protofibrils. The color key on the left shows the local structural

resolution in angstroms (Å) and the colored map indicates the local resolution ranging from 2.4 to 3.8 Å. (**D)** Gold-standard Fourier shell correlation curve. Gold-standard refinement was used for the estimation of the density map resolution. The global resolution of 2.61 Å was calculated using a Fourier shell correlation (FSC) curve cut-off at 0.143.
