# Supplementary material for: Structural basis of a distinct α-synuclein strain that promotes tau inclusion in neurons
Source: J Biol Chem. 2025 Feb 25;301(4):108351. doi: 10.1016/j.jbc.2025.108351 (PMC11982472; doi:10.1016/j.jbc.2025.108351)
Supplement: Figure S4 [file mmc4.pdf]

Figure S4

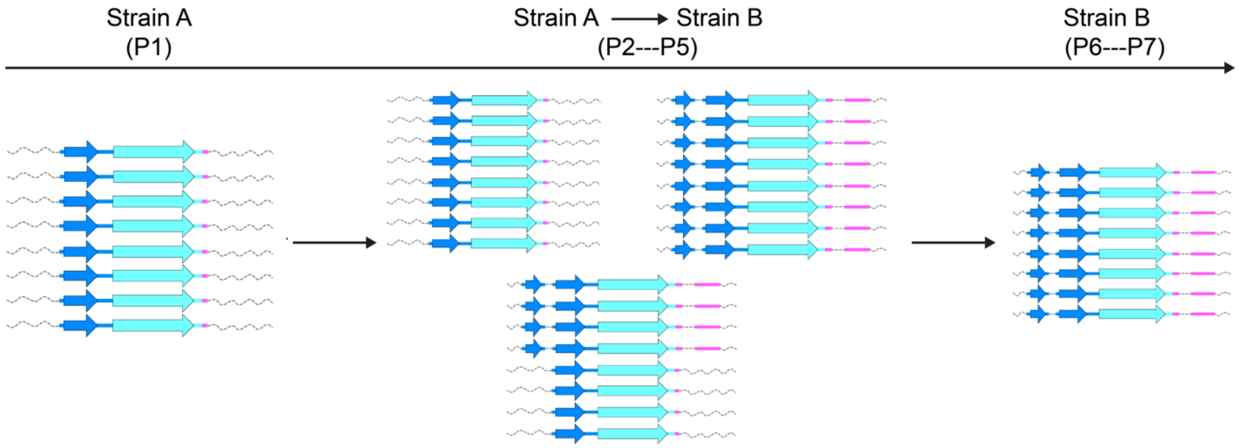

Figure S4. Schematic representation of the generation of strain B fibrils. The schematic diagram shows the possible process of strain B generation. As a-syn monomers are continuously inoculated by different generations of strain, more and more domains are involved in the formation of fibril core.
