# Supplementary material for: Structural basis of a distinct α-synuclein strain that promotes tau inclusion in neurons
Source: J Biol Chem. 2025 Feb 25;301(4):108351. doi: 10.1016/j.jbc.2025.108351 (PMC11982472; doi:10.1016/j.jbc.2025.108351)
Supplement: Figure S5 [file mmc5.pdf]

Figure S5

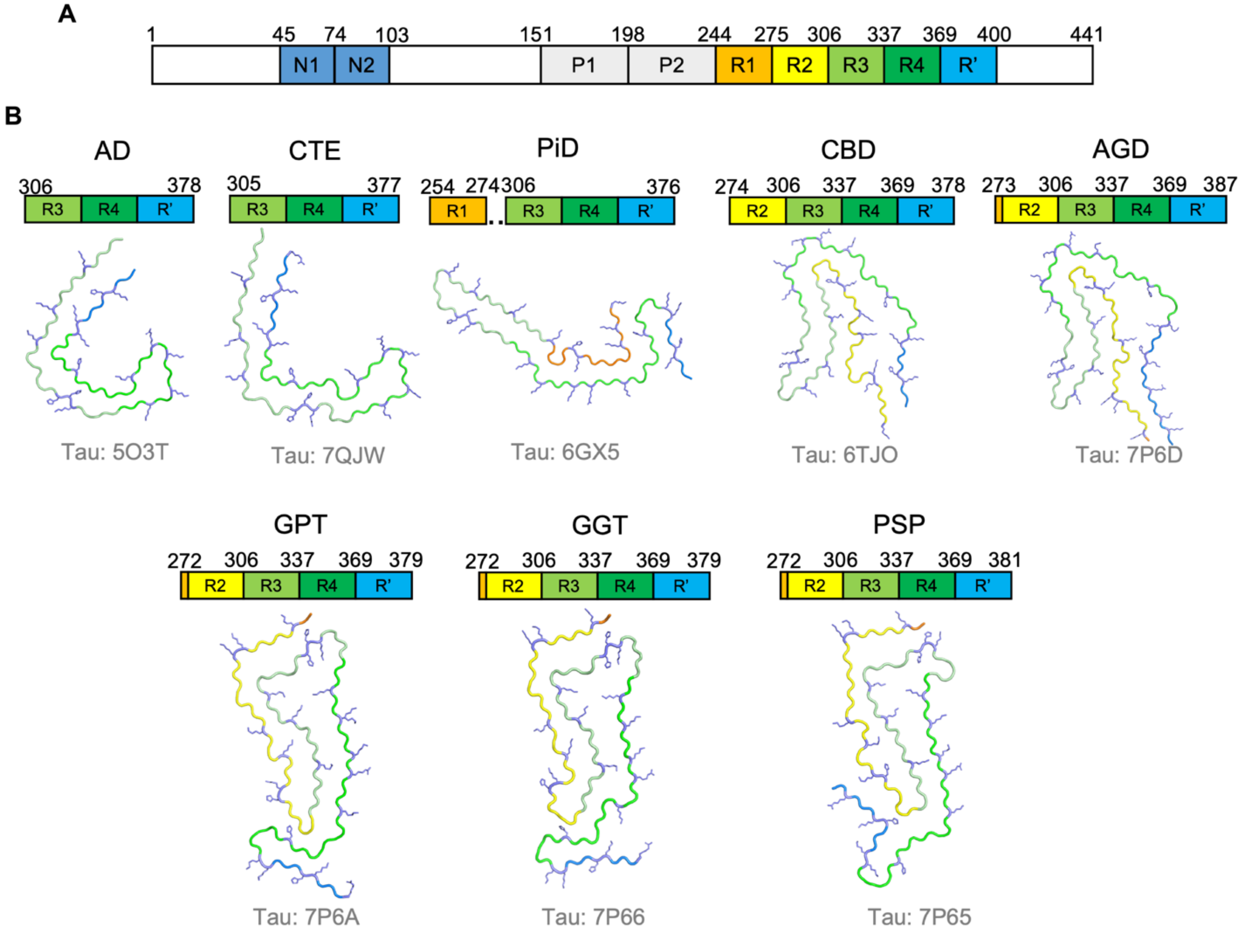

Figure S5. Representative conformations of Tau. (A) Illustrations of tau 2N4R isoform. The 2N4R isoform contains two N-terminal inserts (N1 and N2), two proline-rich regions (P1 and P2), and four microtubule-binding repeats (R1, R2, R3, and R4). The repeat-like segment R' in the C-terminus is also indicated. (**B)** Polymorphic ribbon structures of tau derived from patients with different tauopathies, colors denote different Tau domains (Residues in R1–R4 and R' in the C-terminal domain are colored orange, yellow, pale green, green, and marine, respectively). Positively charged residues in polymorphic forms display as a stick, colored as slate.
