# Supplementary material for: Structural basis of a distinct α-synuclein strain that promotes tau inclusion in neurons
Source: J Biol Chem. 2025 Feb 25;301(4):108351. doi: 10.1016/j.jbc.2025.108351 (PMC11982472; doi:10.1016/j.jbc.2025.108351)
Supplement: Figure S6 [file mmc6.pdf]

Figure S6

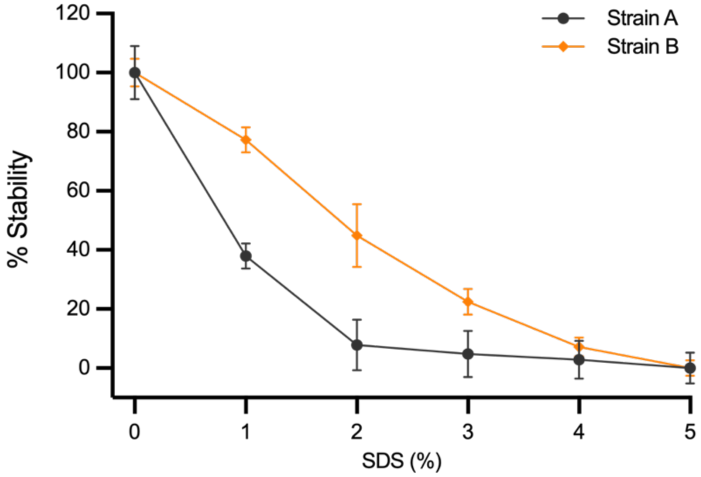

Figure S6. Stability assay of strain A and strain B fibrils. Strain A and strain B fibrils were heated to 37 ˚C and incubated with varying concentrations of SDS. Strain A fibrils show more instability to SDS than strain B fibrils. Individual triplicate measurements are shown, and the plotted line represents the average of the triplicates. Data are shown as mean ± s.d., n = 3 independent experiments.
