# Supplementary material for: Structural basis of a distinct α-synuclein strain that promotes tau inclusion in neurons
Source: J Biol Chem. 2025 Feb 25;301(4):108351. doi: 10.1016/j.jbc.2025.108351 (PMC11982472; doi:10.1016/j.jbc.2025.108351)
Supplement: Figure S7 [file mmc7.pdf]

Figure S7

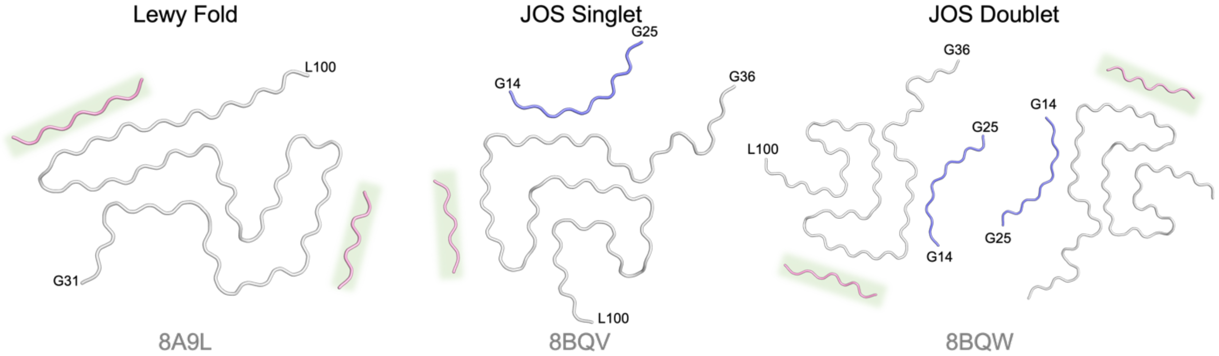

Figure S7. Schematic representation of the cryo-EM structures of amyloid fibrils extracted from patient brains. The figure displays three fibril structures: one from Parkinson's disease (left) and two polymorphs from juvenile-onset synucleinopathy (JOS) (center and right). Unidentified peptide sequences are indicated by green squares.
