# Supplementary material for: Structural basis of a distinct α-synuclein strain that promotes tau inclusion in neurons
Source: J Biol Chem. 2025 Feb 25;301(4):108351. doi: 10.1016/j.jbc.2025.108351 (PMC11982472; doi:10.1016/j.jbc.2025.108351)
Supplement: Figure S8 [file mmc8.pdf]

Figure S8

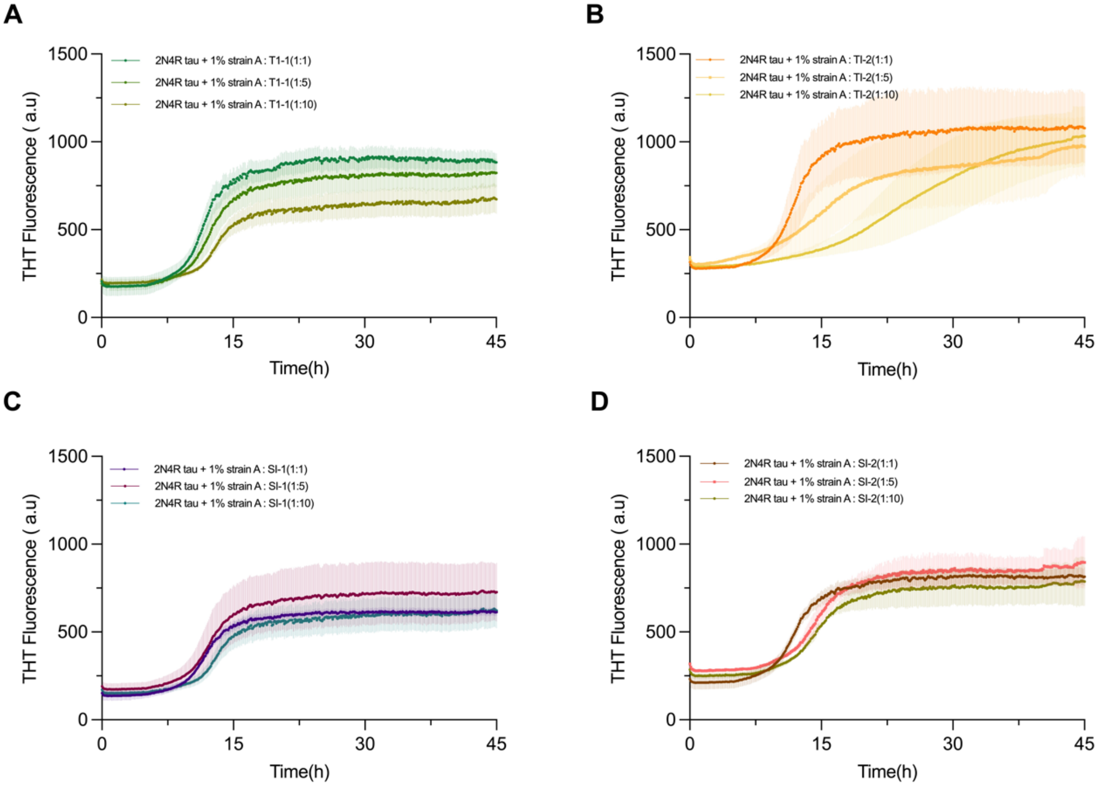

Figure S8. The effect of inhibitors on tau aggregation, as measured by Thioflavin T (ThT) fluorescence. (**A-D**) The impact of different inhibitors and strain A concentrations on tau protein aggregation. Aggregation reactions were performed with 50 µM tau monomers in the presence of 1% strain A and varying inhibitor concentrations (strain A:inhibitor molar ratios of 1:1, 1:5, and 1:10). None of the four inhibitors tested significantly reduced ThT fluorescence. Each curve represents the mean of three independent experiments, with error bars indicating the standard deviation.
