# Supplementary material for: Structural basis of a distinct α-synuclein strain that promotes tau inclusion in neurons
Source: J Biol Chem. 2025 Feb 25;301(4):108351. doi: 10.1016/j.jbc.2025.108351 (PMC11982472; doi:10.1016/j.jbc.2025.108351)
Supplement: Figure S9 [file mmc9.pdf]

Figure S9

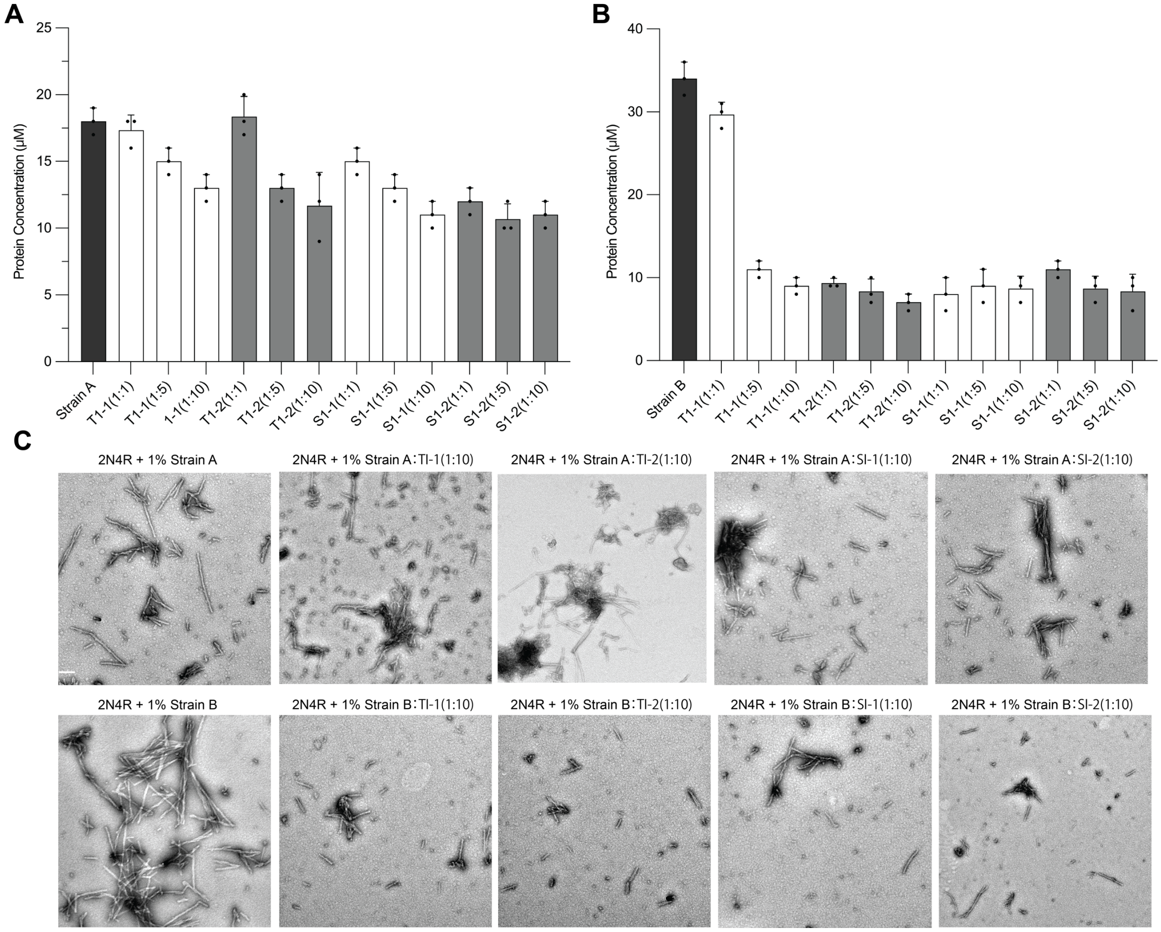

Figure S9. Concentration and morphological profiling of tau protein fibrils under different strains and inhibitor conditions. (**A-B**) Quantitative analysis of tau fibril during the stationary phase of ThT fluorescence. Tau fibrils were pelleted by ultracentrifugation to separate monomeric and fibrillar tau, with protein quantified by the Lowry method. The inhibitor showed no significant effect on Strain A-induced tau aggregation (**A**) but significantly affected Strain B cross-seeding (**B**). (**C**) TEM images of tau variants in different conditions. The scale bar represents 200 nm.
