# Supplementary material for: Structural basis of a distinct α-synuclein strain that promotes tau inclusion in neurons
Source: J Biol Chem. 2025 Feb 25;301(4):108351. doi: 10.1016/j.jbc.2025.108351 (PMC11982472; doi:10.1016/j.jbc.2025.108351)
Supplement: Supplementary Table 1 [file mmc10.docx]

**Supplementary Table 1.** Cryo-EM data collection, refinement, and validation statistics.

|  | **Strain B** |
| --- | --- |
| **Data collection** |  |
| EM equipment | Titan Krios |
| Voltage (KV) | 300 |
| Detector | K3 |
| Nominal Magnification | ×81,000 |
| Pixel size (Å) | 1.079 |
| Total electron dose (e^-^/Å^2^) | 48 |
| Frame exposure time (s) | 0.055 |
| No. of movie frames | 65 |
| Defocus range (μm) | -0.9 ~ -2.2 |
| **Reconstruction** |  |
| No. of micrographs collected | 4,095 |
| No. of micrographs used | 2,934 |
| No. of filaments picked | 46,626 |
| No. of segments for 3D auto-refine | 26,051 |
| Resolution (Å) | 2.61 |
| Map sharpening B-factors (Å^2^) | -74.24 |
| Symmetry for final map | C1 |
| Helical twist (°) | 179.477 |
| Helical rise (Å) | 2.408 |
| **Atomic model** |  |
| No. of atoms | 5,840 |
| No. of protein residues | 840 |
| B-factors (Å^2^) | 53.18 |
| R.m.s deviations |  |
| Bonds length (Å) | 0.006 |
| Bonds angle (°) | 0.789 |
| Ramachandran plot statistics (%) |  |
| Preferred | 92.31 |
| Allowed | 7.69 |
| Outlier | 0 |
| Rotamers outliers (%) | 0 |
| C-beta deviations (%) | 0 |
| Bad bonds (%) | 0 |
| Bad angles (%) | 0 |
| MolProbity score | 1.86 |
| Clash score | 7.01 |
| PDB code | 9C5R |
| EMDB code | EMD-45221 |
